# Engineering Next-Generation Wound Gels for Advanced Healing

**DOI:** 10.3390/gels12100908

**Published:** 2026-10-09

**Authors:** Kannan Badri Narayanan

**Affiliations:** School of Chemical Engineering, Yeungnam University, 280 Daehak-Ro, Gyeongsan 38541, Republic of Korea; okbadri@gmail.com or okbadri@yu.ac.kr

## 1. Introduction

Wound healing is frequently perceived as a straightforward biological process; in practice, however, it remains a major challenge in clinical medicine due to the intricate, overlapping phases of hemostasis, inflammation, proliferation, and tissue remodeling [1]. The failure of any single phase to resolve appropriately can result in delayed wound closure, chronic ulceration, or infection, outcomes that impose a substantial burden on global healthcare systems. This burden is further exacerbated by an aging population, the rising prevalence of diabetes, and the progressive decline in antibiotic efficacy against biofilm-forming pathogens. Conventional wound dressing materials, including gauze, films, and simple hydrocolloids, function principally as passive physical barriers, offering protection from the external environment while contributing little to the active biochemical signaling required for tissue repair. Despite their capacity to maintain hydration and provide mechanical protection, these materials frequently underperform clinically because of their static nature, susceptibility to biofilm formation, inconsistent drug release kinetics, and vulnerability to multidrug-resistant (MDR) pathogens [2,3,4]. These inherent limitations have driven a paradigm shift toward the design of dynamic, bioresponsive wound dressings that actively participate in the healing process rather than merely cover the wound, specifically smart biomaterials capable of sensing and responding to, rather than simply shielding, the local wound microenvironment.

Hydrogels are three-dimensional (3D) hydrophilic polymer networks capable of retaining substantial water content while maintaining structural integrity; they have emerged as the ideal polymeric matrix for these broader applications. Their tissue-like mechanical compliance, tunable porosity, and compatibility with a broad range of natural and synthetic polymer chemistries allow them to be engineered as multifunctional systems capable of regulating moisture balance, delivering therapeutic payloads, resisting microbial colonization, and, in the most sophisticated formulations, sensing and dynamically responding to local biochemical cues. Contemporary research focuses on replicating the native wound microenvironment through such intelligent, bioinspired hydrogel platforms, which incorporate adaptable structural features such as tunable stiffness, biochemical gradients, and stimulus-responsive release of therapeutic agents. Clinical viability, however, requires careful optimization of parameters, including oxygen and glucose diffusion, porosity, and construct architecture, and computer-aided design (CAD) approaches have been increasingly used to more closely recapitulate the complexity of native tissue [5]. The field is advancing further toward multi-scale additive manufacturing (MSAM) and 4D bioprinting, which enable hydrogel constructs to undergo post-fabrication temporal transformations, such as controlled swelling, contraction, or self-assembly into vascular-like networks, in response to biochemical gradients [6,7]. Concurrently, the incorporation of non-cytotoxic quorum-sensing inhibitors and bacteriophage-based scaffolds provides additional defense against biofilm formation and MDR pathogens, extending the clinical relevance and translational potential of these systems [4].

This Special Issue, “*Designing Gels for Wound Dressing (2nd Edition)*”, extends this paradigm, bringing together eight contributions (comprising five original research studies, two comprehensive reviews, and one methodological study) that, collectively, illustrate the mechanisms by which contemporary hydrogel science is converging on several parallel design strategies. The first strategy is the reconciliation of mechanical toughness with biological activity through interpenetrating and dual-network architectures. The second strategy is the exploitation of photochemical and other tunable crosslinking chemistries to control network density, degradation, and secondary bioactivity. The third strategy is the incorporation of increasingly complex bioactive agents, ranging from small-molecule phytochemicals and platelet agonists to complex, multicomponent cell secretomes. Finally, the fourth strategy is the integration of advanced biofabrication platforms, particularly 3D bioprinting, which enable spatial control over construct architecture and payload distribution.

## 2. Overview of the Papers Published in This Issue

The second edition of the Special Issue “Designing Gels for Wound Dressing” comprises eight contributions that collectively address the integration of bioactivity, material formulation, and manufacturing methodologies to optimize wound healing. These contributions converge across four thematic domains. The first domain addresses polymer network architecture, wherein interpenetrating and dual-network strategies reconcile the competing requirements of mechanical toughness and biological activity [8,9]. The second domain focuses on crosslinking chemistry, in which photoinitiated and stimuli-responsive crosslinking systems enable rapid, spatially controllable gelation while imparting secondary functionalities, such as antimicrobial activity [10,11]. The third domain encompasses bioactive therapeutic payload delivery, spanning a spectrum from small-molecule therapeutics and platelet agonists [12] to complex, multicomponent biological secretomes [13]. The fourth domain is addressed by two complementary contributions, specifically Narayanan’s [14] review of scaffold-based and scaffold-free 3D bioprinting in tissue engineering and regenerative medicine, alongside Carmona and Lopez’s [15] methodological analysis of platelet-rich gel supernatant (PRGS) stability. Collectively, these studies demonstrate the advanced fabrication platforms and quality control frameworks necessary to translate laboratory-scale innovations into reproducible, clinically viable products. Overall, the findings reveal an integrated design strategy in which next-generation wound dressings are increasingly engineered as interconnected systems that synergistically combine mechanical scaffolding, chemical crosslinking, and biological signaling, rather than functioning as independent or additive components.

### 2.1. Key Highlights from Research Articles

#### 2.1.1. Reconciliation of Mechanical Toughness and Bioactivity Through Interpenetrating Networks

A persistent limitation of polysaccharide-based hydrogels has been their relatively poor mechanical performance, which restricts their structural integrity and operational durability at wound sites subjected to movement and exudate pressure. Li et al. [8] addressed this constraint by constructing a dual-network hydrogel composed of Salecan (SA), a bacterial exopolysaccharide, and polyvinyl alcohol (PVA). In their study, a primary network assembled through electrostatic interactions between polydopamine (PDA) and Salecan served as a template for the *in situ* reduction of silver nitrate into silver nanoparticles (AgNPs). A secondary PVA network, consolidated through physical crosslinking via repeated freeze–thaw cycles, provided additional structural reinforcement. The resulting SA/PDA/AgNPs/PVA composite hydrogel achieved a tensile strength of 0.31 MPa, an elongation at break of 158.9%, and a fracture toughness of 31.16 J·m^−2^, demonstrating substantially enhanced biomechanical properties. Antibacterial assays revealed inhibition rates of 99.5% against *Staphylococcus aureus* and 80.1% against *Escherichia coli.* Furthermore, in a murine infected-wound model, near-complete closure with minimal scarring was observed within 12 days, accompanied by upregulated CD31 expression indicative of enhanced neovascularization. In addition, the PDA component conferred robust tissue adhesion by forming multiple hydrogen bonds with the cutaneous skin surface, a property that supports the wound dressing performance of the SA/PDA/AgNPs/PVA composite hydrogel in promoting the healing of infected, chronic wounds [8].

A complementary strategy was reported by Chen et al. [9], who fabricated a multifunctional silver-loaded hydrogel film dressing from a transparent, elastic PVA/chitosan (PVA/CS) hybrid matrix incorporating AgNPs synthesized using a redox reduction method to maintain a sterile wound microenvironment over an extended period. Beyond its broad-spectrum antibacterial activity, which is mediated by bacterial membrane disruption, genomic DNA damage, and reactive oxygen species (ROS) generation characteristic of AgNPs, this hydrogel film achieved an optical transmittance exceeding 75.02%, alongside favorable swelling kinetics, robust mechanical properties, and substantial protein adsorption capacity. Drug loading and drug release experiments demonstrated high encapsulation efficiency and sustained release profiles. Both the cumulative release amount and the duration required for complete release varied as a function of AgNP content across different pH conditions, confirming pH-responsive release behavior. Together, these two studies demonstrate that mechanical toughness, antimicrobial defense, and sustained drug delivery can be synergistically integrated within a single polymeric matrix rather than engineered as independent parameters [9].

#### 2.1.2. Photocrosslinking as a Tool for Tunable Network Architecture

Photopolymerization provides a rapid, *in situ* gelation mechanism that affords precise control over polymer network density. Two studies in this Special Issue utilized this strategy for targeted biomedical applications. Nacu et al. [10] synthesized hydrogel dressings by combining methacrylated chitosan with methacrylated gelatin or allylic gelatin in varying formulations, followed by photocrosslinking using Irgacure 2959 as a photoinitiator. The allylic gelatin-based hydrogels exhibited larger pores and stronger pH-responsive swelling behavior relative to methacrylated gelatin hydrogels, reflecting the higher flexibility of the allylic gelatin network. These hydrogels displayed sustained bacitracin elution over an initial 6 h observation period via non-Fickian diffusion mechanisms. Enhanced bioadhesive properties were exhibited for the more flexible allylic gelatin formulations, increasing their suitability for wound management. Importantly, cytocompatibility across two independent fibroblast cell lines (normal human dermal fibroblasts (NHDFs) and BALB/3T3) was preserved across all formulations, and *in vitro* scratch assays confirmed accelerated cell migration and gap closure relative to untreated controls. Thus, combining these biopolymers with targeted chemical modifications yields versatile network architectures optimized for controlled drug delivery and tissue regeneration [10].

Gelatin-based hydrogels are promising matrices for wound management. A novel approach to photocrosslinking gelatin hydrogels was presented by Abramov et al. [11], who utilized menadione sodium bisulfite (MSB), a water-soluble hemostatic derivative of vitamin K3, as a multifunctional photoinitiator, hemostatic agent, and antimicrobial additive within porcine and fish gelatin matrices. Upon ultraviolet (UV) irradiation, MSB transitions to a reactive triplet state wherein its carbonyl oxygen acts as a hydrogen-abstracting radical acceptor, operating via a mechanism analogous to quinone- and ketone-based photoinitiators. The excited MSB molecule preferentially abstracts hydrogen atoms from α-carbon sites along the gelatin backbone and amino acid side chains, forming carbon-centered protein radicals. Interchain radical recombination subsequently generates covalent carbon–carbon (C–C) crosslinks, stabilizing the gelatin matrix. MSB-mediated crosslinking increased antimicrobial efficacy against *S. aureus*, *C. albicans*, and *E. coli* by up to 5.6-fold relative to noncrosslinked control hydrogels. Integrating matrix stabilization with intrinsic antimicrobial and hemostatic capabilities establishes these MSB-crosslinked hydrogels as promising candidates for multifunctional wound dressings [11].

#### 2.1.3. Multifunctional Hydrogels Modulating Wound Repair Processes

The development of a single multifunctional wound dressing that integrates rapid hemostatic function with antibacterial, antioxidant, and anti-inflammatory properties represents a significant advance in wound dressing materials. Abbas et al. [12] developed a novel multifunctional gelatin methacryloyl (GelMA) hydrogel co-loaded with curcumin (Cur) and adenosine diphosphate (ADP). This platform synergizes the antibacterial, antioxidant, and anti-inflammatory activities of curcumin with the pro-coagulant and immunomodulatory functions of ADP within a biocompatible matrix for traumatic wound management. The Cur/ADP–GelMA hydrogels exhibited robust tissue adhesion, structural stability, and broad-spectrum antibacterial activity, suppressing *E. coli* and *S. aureus* proliferation by 85% and 72%, respectively. Furthermore, in a rat tail amputation model, the hydrogels demonstrated rapid hemostasis, decreasing total blood loss to 238 ± 23 mg compared to 559 ± 18 mg in untreated control groups. This hemostatic efficacy is attributed to ADP-mediated activation of platelet P2Y_1_ and P2Y_12_ purinergic receptors. Enzyme-linked immunosorbent assay (ELISA) quantifications confirmed significant downregulation of pro-inflammatory cytokines, specifically tumor necrosis factor-alpha (TNF-α), interleukin-1 beta (IL-1β), and interleukin-6 (IL-6), alongside concurrent upregulation of anti-inflammatory interleukin-10 (IL-10). In addition, *in vivo* evaluations in a murine full-thickness wound model demonstrated 98% wound closure by day 14, enhanced granulation tissue maturation, and a 70% increase in epidermal thickness relative to control cohorts. Mechanistically, ADP accelerates platelet activation and fibrin clot formation during early hemostasis, while curcumin modulates the inflammatory microenvironment, facilitating synergistic immune regulation and accelerated tissue regeneration [12].

#### 2.1.4. Beyond Synthetic Hydrogels and Standardizing Biologically Derived Matrices

Platelet-rich gel supernatants (PRGSs) contain a concentrated mixture of anabolic growth factors, including platelet-derived growth factors (PDGFs), vascular endothelial growth factor (VEGF), transforming growth factor beta-1 (TGF-β1), epidermal growth factor (EGF), and basic fibroblast growth factor (bFGF), alongside anti-inflammatory cytokines, specifically interleukin-1 receptor antagonist (IL-1ra) and IL-4, as well as cell-adhesive glycoproteins such as fibronectin and vitronectin. These bioactive constituents function synergistically to promote cell proliferation, migration, neoangiogenesis, and accelerated tissue regeneration [16]. A methodologically distinct yet conceptually complementary investigation was conducted by Carmona and Lopez [15], who applied multivariate statistical analyses to characterize the integrated inflammatory and regenerative signatures of bovine PRGS stored across different temperature range conditions (–80 °C, –20 °C, 4 °C, 21 °C, and 37 °C) over incubation periods extending up to 326 h. The TGF-β1, TNF-α, IL-2, and IL-6 in PRGS samples collected from six clinically healthy bovine donors were quantified and evaluated over the 326 h observation period. Standardized datasets were evaluated using hierarchical cluster analysis, heatmaps, and principal component analysis (PCA) derived from concentration-versus-time area under the curve (AUC) metrics. Formulations maintained under sub-zero storage conditions (–20 °C and –80 °C) clustered independently from those kept at ambient or physiological temperatures. The first two principal components, PC1 and PC2, accounted for 43.0% and 28.9% of the total variance, respectively, establishing an inflammatory-to-regenerative signaling gradient that contrasted TGF-β1 and IL-2 expression against TNF-α and IL-6 profiles. Linear mixed-effects modeling confirmed significant temperature- and time-dependent variations across both principal components (*p* ≤ 0.048). Thermal divergence increased after 48 to 96 h of storage, demonstrating that environmental temperature fundamentally alters the integrated biological profile of PRGS.

### 2.2. Insights from Review Articles

#### 2.2.1. Secretome-Loaded Biomaterials as a Bridge Between Structural and Signaling Paradigms

The cell secretome comprises a complex mixture of biologically active molecules secreted into the extracellular environment, including growth factors, cytokines, chemokines, functional proteins, enzymes, and extracellular vesicles (EVs). Often conceptualized as the paracrine signaling machinery of the cell, the secretome mediates intercellular communication and alters local tissue microenvironments. The therapeutic efficacy of the secretome stems from its multifactorial and coordinated paracrine activity, which enables the simultaneous orchestration of inflammatory responses, angiogenesis, cell proliferation, cell migration, ECM remodeling, and tissue maturation. These collective properties establish secretome-based formulations as highly promising therapeutic platforms for wound repair and regenerative medicine [17]. While conventional biomaterials provide structural scaffolding for tissue reconstruction, they often lack the biochemical signaling cues required for active tissue regeneration. Incorporating cell-secreted factors into biomaterial matrices addresses this limitation, yielding secretome-loaded biomaterials capable of actively directing repair processes. Rattanachot et al. [13] provided a comprehensive review of secretome-loaded three-dimensional (3D) printed biomaterials, as the collective cytokines, growth factors, and extracellular vesicles secreted by cultured cells are an advantageous alternative to traditional biomaterials loaded with single recombinant growth factors. Their review detailed extrusion-based and advanced 3D bioprinting modalities that facilitate the spatially controlled deposition of secretome-laden bioinks. These engineered constructs modulate inflammation, stimulate neoangiogenesis, and support ECM remodeling in a physiologically coordinated manner. Finally, the authors addressed ongoing translational bottlenecks, including secretome collection standardization, batch-to-batch reproducibility, and complex regulatory approval pathways, such as achieving compliance with Good Manufacturing Practice (GMP) standards for clinical translation in wound management [13].

#### 2.2.2. Bioprinting Modalities and the Architecture of Regenerative Constructs

Because human skin is a complex, multilayered tissue, conventional wound care approaches cannot replicate its native anatomical architecture. Next-generation 3D bioprinting allows the precise spatial deposition of cells, structural biomaterials, growth factors, and bioactive molecules to construct functional living skin tissues for accelerated wound closure [18]. The comprehensive review by Narayanan [14] evaluates recent advances in scaffold-based and scaffold-free 3D bioprinting strategies across diverse tissue targets, including skin, bone, cartilage, and vascular systems. The review highlights various applications of extrusion-based, droplet-based, laser-assisted, and light-based bioprinting modalities in tissue engineering. Light-based 3D bioprinting, particularly two-photon polymerization (2PP), has emerged as a crucial tool due to its ability to fabricate biomimetic ECM architectures and intricate microvascular networks with sub-micron spatial resolution. In contrast, volumetric bioprinting (VBP) polymerizes entire 3D constructs within a rotating photosensitive bioresin via tomographic light projections, enabling the fabrication of centimeter-scale, cell-laden structures within seconds while preserving high cell viability. Narayanan [14] also addresses scaffold-free 3D bioprinting and bioassembly techniques, such as the Kenzan method, aspiration-assisted bioprinting, and magnetic field-directed assembly, which facilitate the construction of spheroid- and organoid-based tissues without exogenous polymeric carriers. Notably, the review emphasizes that the physical printability of a bioink is distinct from its biological functionality. Printability denotes the capacity of a bioink or precursor solution to maintain structural fidelity and shape retention post-fabrication. Conversely, biological performance depends on its ability to support cell survival, proliferation, lineage-specific differentiation, tissue integration, and functional wound regeneration. This distinction is paramount for both scaffold-based and scaffold-free 3D bioprinting platforms. Consequently, optimizing physical properties, such as rheological behavior, mechanical strength, porosity, and swelling kinetics, must be carefully balanced with biological functionality. Ultimately, the efficacy of bioprinted wound constructs should be evaluated by not only structural fidelity but also their capacity to promote functional tissue repair in physiologically relevant *in vitro* and *in vivo* wound models [14].

## 3. Methodological Limitations and Translational Challenges

Despite the notable technical innovations presented across this Special Issue, several methodological limitations persist that require systematic resolution, as they collectively represent the translation bottlenecks governing clinical adoption.

1. Reliance on small-animal and short-duration models: The *in vivo* validation reported across the included studies predominantly relies on acute rodent models evaluated over short observation periods. For instance, Li et al. [8] demonstrated near-complete tissue repair of *S. aureus*-infected wounds within 12 days using a murine model, while Abbas et al. [12] reported 98% re-epithelialization by day 14 in a similar acute small-animal murine model. Although such models are indispensable for establishing baseline therapeutic efficacy, rodent cutaneous wound closure is largely contraction-driven, mediated by myofibroblasts and the panniculus carnosus (a subcutaneous muscle layer present in rodents). As the wound contracts, myofibroblasts generate force and remodel the ECM, which dominates the early to mid-phases of healing in small wounds. This contrast to humans is well-established; human wound healing is less dependent on contraction and is instead dominated by the re-epithelialization and granulation tissue remodeling characteristic of human skin [19]. Furthermore, none of the featured studies evaluated translational performance in large-animal models or impaired healing microenvironments, such as diabetic or ischemic ulceration, which can alter the balance of healing processes. Therefore, although high percentages of re-epithelialization by day 14 can be observed in some murine wounds, extrapolating these data to human healing requires validation from translational models, including splinted wounds, diabetic models, or large-animal studies. Abramov et al. [11] explicitly delineated these translational constraints, stating that the structural and antimicrobial characterizations of MSB–crosslinked gelatin hydrogels require subsequent, multi-parametric validations of cytocompatibility, biomechanical stability under physiological fluid loads, and mature biofilm eradication prior to clinical advancement.
2. Persistent concerns over nanoparticle and additive safety: Incorporating silver nanoparticles (AgNPs), investigated discretely by Chen et al. [9] and Li et al. [8], remains the primary antimicrobial strategy reported in this Special Issue. However, neither study evaluated long-term systemic silver bioaccumulation, transdermal permeation kinetics, or chronic cytotoxicity beyond the acute wound healing phase. Similarly, although Abbas et al. [12] performed histological evaluations of major organs to assess biocompatibility, the evaluation phase was restricted to a 14-day observation period. This short experimental duration perpetuates unresolved questions regarding cumulative toxicological exposure during prolonged dressing application on chronic wounds. This gap reflects a discrepancy in the antimicrobial hydrogel literature between demonstrated acute therapeutic efficacy and the rigorous, long-term biocompatibility and safety profiles required for regulatory approval.
3. Batch-to-batch variability and reproducibility of biologically derived and *in situ*-synthesized components: Two studies in this Special Issue exemplify these translational bottlenecks. Rattanachot et al. [13] demonstrated that secretome composition varies depending on donor cell source, passage number, and harvesting protocol, thereby complicating the standardization required for consistent therapeutic dosing. Furthermore, the authors indicated that secretome-loaded bioprinted constructs present a complex regulatory classification between medical devices and biological products, a designation that complicates regulatory clearance pathways even when compliance with Good Manufacturing Practice (GMP) standards is achieved. Carmona and Lopez [15] substantiated these concerns using a multivariate analysis of bovine platelet-rich gel supernatants (PRGSs). They demonstrated that storage temperature and duration modulate the coordinated inflammatory and regenerative signaling signatures of the biological matrix as an integrated system, rather than exclusively degrading individual growth factors. This degradation implies that quality control frameworks relying on single-analyte potency assays are insufficient for characterizing complex biological payloads. Similarly, *in situ* nanoparticle synthesis, demonstrated by Li et al. [8] via polydopamine-mediated reduction of silver ions, introduces another source of batch-to-batch variability. Nanoparticle size distribution, morphology, and yield remain highly sensitive to local reaction kinetics, which are intrinsically more challenging to standardize than pre-synthesized nanoparticle formulations.
4. The interplay between network crosslinking density and biological performance: Several studies in this Special Issue illustrate that structural parameters optimized to enhance mechanical integrity or barrier function often impair competing functional properties. Nacu et al. [10] demonstrated that increasing methacrylation-derived crosslinking density in photocrosslinked chitosan/gelatin hydrogels reduced network porosity, thereby diminishing the cumulative release kinetics of the model drug bacitracin. This finding demonstrates an inherent structural trade-off between biomechanical reinforcement and therapeutic payload delivery. Abramov et al. [11] reported an analogous structure–function compromise regarding the thermal stabilization of collagen-like triple helices. Although MSB-mediated crosslinking enhanced antimicrobial efficacy, the photochemical nature of the reaction presents persistent limitations concerning light penetration depth and crosslinking uniformity within thicker hydrogel constructs. Collectively, these findings indicate that optimizing a single hydrogel property independently frequently compromises overall functional performance, emphasizing the critical need for multi-objective material design frameworks over sequential, single-parameter optimization approaches.
5. Scalability and resolution constraints in advanced biofabrication: Narayanan [14] highlighted substantial advances in extrusion-, inkjet-, laser-assisted, and light-based bioprinting, as well as emerging techniques such as Freeform Reversible Embedding of suspended Hydrogels (FRESH) and volumetric bioprinting. Despite these technological advances, no single bioprinting platform or bioink formulation currently provides a universal solution capable of simultaneously achieving high resolution, scalability, structural fidelity, and biological functionality. Constructs satisfying geometric printability criteria often prove physiologically non-functional if downstream biological metrics, such as lineage-appropriate differentiation or epidermal barrier integrity in engineered skin constructs, are not independently validated. This distinction between morphological fidelity and physiological functionality represents a broader conceptual challenge that extends beyond biofabrication to the field of hydrogel-based wound dressings, where swelling behavior and mechanical properties are often reported without corresponding functional validation in physiologically relevant wound models.

## 4. Conclusions and Future Perspectives

Taken together, the contributions to “Designing Gels for Wound Dressing (2nd Edition)” demonstrate a clear progression in which wound dressings are evolving from passive physical barriers into integrated, multifunctional therapeutic platforms. These advanced fabrication platforms combine robust mechanical properties, tunable crosslinking chemistries, and biologically coordinated payloads. The interpenetrating polymer network (IPN) strategies reported by Li et al. [8] and Chen et al. [9] demonstrate that natural–polymer hydrogels can achieve mechanical profiles suitable for demanding clinical applications. Photocrosslinking innovations introduced by Nacu et al. [10] and Abramov et al. [11] establish that crosslinking chemistries can be engineered to impart secondary therapeutic functions, such as tunable pore architectures and intrinsic antimicrobial activity. Furthermore, the dual-payload formulation presented by Abbas et al. [12] highlights the clinical efficacy of temporally sequenced multi-agent drug release. Complementing these experimental advances, the review articles and the methodological framework established by Carmona and Lopez [15] provide the biofabrication and quality control frameworks required to translate these complex formulations into reproducible clinical products.

Consequently, several critical areas demand systematic investigation to facilitate clinical translation. First, spatially and temporally patterned biofabrication: the integration of light-based and volumetric bioprinting techniques, reviewed by Narayanan [14], with multifunctional bioink chemistries points toward near-term developments in spatially patterned wound dressings. Concurrently, emerging techniques such as stereolithography apparatus for tissue engineering (SLATE), bioprinting-assisted tissue emergence (BATE), and embedding bioprinting are advancing 3D biofabrication across both scaffold-based and scaffold-free platforms [20]. Such constructs potentially enable region-specific, kinetically controlled release of distinct bioactive agents tailored to the localized healing stages across a heterogeneous wound bed. Second, complex biological payloads require standardization: harnessing cell secretomes, as discussed by Rattanachot et al. [13], requires overcoming batch-to-batch variability and regulatory hurdles. Developing standardized secretome characterization protocols that leverage multivariate, systems-level analytical tools, similar to those applied by Carmona and Lopez to platelet-derived matrices, will be essential to ensure quality control comparable to synthetic hydrogel platforms [15]. Third, pharmacologically active crosslinkers represent ideal candidates for repurposing: the successful utilization of clinically approved small molecules, such as menadione sodium bisulfite (MSB), as dual-function crosslinking and antimicrobial agents encourages the systematic exploration of other therapeutic molecules. Repurposing active compounds as structural network elements simplifies overall formulation complexity while broadening biological functionality. Fourth, translational gaps must be addressed: to achieve successful clinical translation, studies must address recurring methodological constraints, and preclinical validation must extend beyond acute small-animal models to include large-animal systems and chronic, impaired-healing microenvironments, such as diabetic or ischemic ulcers, while long-term toxicological evaluations for biomaterials containing nanoparticles and photoinitiators are required alongside robust GMP protocols for biologically derived and *in situ* synthesized components. Overall, the studies compiled in this Special Issue provide the technical foundation and rigorous evaluation required to advance the field of wound dressing development and regenerative medicine. Furthermore, this Special Issue promotes interdisciplinary collaboration across polymer chemistry, regenerative biology, and additive manufacturing, ultimately accelerating the translation of next-generation wound gels from the laboratory bench to clinical practice.
